# Polarity-Specific Cortical Effects of Transcranial Direct Current Stimulation in Primary Somatosensory Cortex of Healthy Humans

**DOI:** 10.3389/fnhum.2016.00208

**Published:** 2016-05-09

**Authors:** Robert Rehmann, Matthias Sczesny-Kaiser, Melanie Lenz, Tomasz Gucia, Annika Schliesing, Peter Schwenkreis, Martin Tegenthoff, Oliver Höffken

**Affiliations:** Department of Neurology, BG-Universitaetsklinikum Bergmannsheil BochumBochum, Germany

**Keywords:** tDCS, primary somatosensory cortex, excitability, neuronal plasticity, humans

## Abstract

Transcranial direct current stimulation (tDCS) is a non-invasive stimulation method that has been shown to modulate the excitability of the motor and visual cortices in human subjects in a polarity dependent manner in previous studies. The aim of our study was to investigate whether anodal and cathodal tDCS can also be used to modulate the excitability of the human primary somatosensory cortex (S1). We measured paired-pulse suppression (PPS) of somatosensory evoked potentials in 36 right-handed volunteers before and after anodal, cathodal, or sham stimulation over the right non-dominant S1. Paired-pulse stimulation of the median nerve was performed at the dominant and non-dominant hand. After anodal tDCS, PPS was reduced in the ipsilateral S1 compared to sham stimulation, indicating an excitatory effect of anodal tDCS. In contrast, PPS in the stimulated left hemisphere was increased after cathodal tDCS, indicating an inhibitory effect of cathodal tDCS. Sham stimulation induced no pre–post differences. Thus, tDCS can be used to modulate the excitability of S1 in polarity-dependent manner, which can be assessed by PPS. An interesting topic for further studies could be the investigation of direct correlations between sensory changes and excitability changes induced by tDCS.

## Introduction

Cortical excitability is regarded as an essential factor contributing to successful perceptual learning. For the somatosensory system, a correlation between the efficiency of perceptual learning and excitability changes of cortical neuronal populations has been described (Höffken et al., 2007). Transcranial direct current stimulation (tDCS) is a non-invasive stimulation method which has been shown in previous studies to modulate the excitability of the motor and visual cortices in human subjects in a polarity dependent manner (Nitsche and Paulus, 2000, 2001; Antal et al., 2003, 2006; Nitsche et al., 2003a, 2008; Moliadze et al., 2014). Cathodal stimulation has been shown to decrease cortical excitability while conversely anodal stimulation enhances it (Nitsche and Paulus, 2000, 2001; Antal et al., 2003; Nitsche et al., 2003a). Animal studies suggest that cathodal tDCS reduces spontaneous firing rates of cortical neurons, most likely by hyperpolarizing neuronal membranes at subthreshold level, while anodal stimulation results in a reversed effect, by depolarizing neurons (Creutzfeldt et al., 1962; Bindman et al., 1964; Ward and Weiskrantz, 1969; Antal et al., 2003, 2004). The manipulation of cortical excitability of the motor and visual system by tDCS influences perception and learning, which has been shown in several studies (Nitsche et al., 2003b; Antal et al., 2004, 2006; Bachmann et al., 2010; Kraft et al., 2010; Bastani and Jaberzadeh, 2012; Zimerman et al., 2013). Several psychophysiological studies suggest that the effect of tDCS of the primary sensory cortex (S1) is similar to the effect found in the motor and visual cortex. Rogalewski et al. (2004) compared tactile discrimination of vibratory stimuli to the left ring finger prior to, during and after cathodal, anodal and sham tDCS over the corresponding somatosensory cortex at C4 according to the 10–20 EEG international system (Jasper, 1958). Cathodal stimulation compared with sham induced a prolonged decrease of tactile discrimination, while anodal and sham stimulation did not. Ragert et al. (2008) demonstrated that a short period of anodal tDCS applied over the human S1 enhances tactile spatial acuity in a grating orientation task in the contralateral hand relative to sham stimulation. A more recent study used quantitative sensory testing to analyze the effects of S1 tDCS on thermal and mechanical perception and demonstrated that cathodal stimulation induced a decrease of Aδ-fiber mediated sensitivity, namely, cold detection at innocuous stimulation intensities (Grundmann et al., 2011). Antal et al. (2008) showed that cathodal tDCS over S1 significantly diminished pain perception and the amplitude of the N2 component when the hand contralateral to the side of tDCS was laser-stimulated, whereas anodal and sham stimulation conditions had no significant effect. Two recent studies assessed the effect of tDCS over the sensorimotor cortex on the amplitudes of somatosensory evoked potentials (SEPs) in humans (Matsunaga et al., 2004; Dieckhöfer et al., 2006). Matsunaga et al. (2004) showed that 1 mA anodal tDCS over the sensorimotor cortex results in long lasting increase in the amplitudes of the P22/N30, P25/N33 and N33/P40 components of the SEP evoked by stimulation of the contralateral, but not ipsilateral median nerve. In the second study (Dieckhöfer et al., 2006) there was a significant reduction of the N20 source amplitude after cathodal tDCS to the somatosensory cortex. These studies support the hypothesis that tDCS can induce excitability changes in S1 in a polarity dependent manner.

The aim of our study was to use paired-pulse stimulation in combination with SEP recordings as a well-established marker of somatosensory cortical excitability (Höffken et al., 2007, 2010, 2013; Lenz et al., 2011, 2012) to investigate whether anodal and cathodal tDCS modulate the excitability of the human somatosensory cortex in the same polarity dependent manner as it was previously described for the motor and visual cortices. We measured paired-pulse suppression (PPS) before and after anodal, cathodal, or sham tDCS stimulation over the right primary somatosensory cortex in healthy right-handed subjects. PPS describes the phenomenon that at short stimulus onset asynchronies (SOAs) neuronal responses to the second stimulus are significantly reduced. PPS is quantified as the ratio of the second response amplitude divided by the first response amplitude. Small amplitude ratios are associated with strong PPS; large ratios are associated with reduced PPS (Höffken et al., 2007, 2010, 2013; Lenz et al., 2011, 2012).

## Materials and Methods

### Subjects

We tested 37 right-handed volunteers aged 19–52 years. One experiment had to be stopped due to defect electrode cable of the tDCS device. The data of this subject was excluded from analysis. The mean age of our remaining 36 subjects was 27.1 ± 7.6 years (18 female, mean age 25.0 ± 7.0 years; 18 male, mean age 29.3 ± 7.8 years). In all subjects, handedness was determined using the Edinburgh Handedness Inventory (Oldfield, 1971). All subjects were neurologically healthy. Individuals with diseases of the central or peripheral nervous system were excluded from the study. Present or past medication with central nervous effects was an additional criterion for exclusion. Exclusion criteria for tDCS were all kinds of metallic implants or electrical devices, active epilepsy and pregnancy. All participants gave written informed consent and were paid for participation.

The study was approved by the Ethics Committee of the Medical Faculty of Ruhr-University of Bochum (Nr. 4309-12) and was performed in accordance to the Declaration of Helsinki.

### Paired-Pulse Evoked Somatosensory Potentials

**FIGURE 1:**
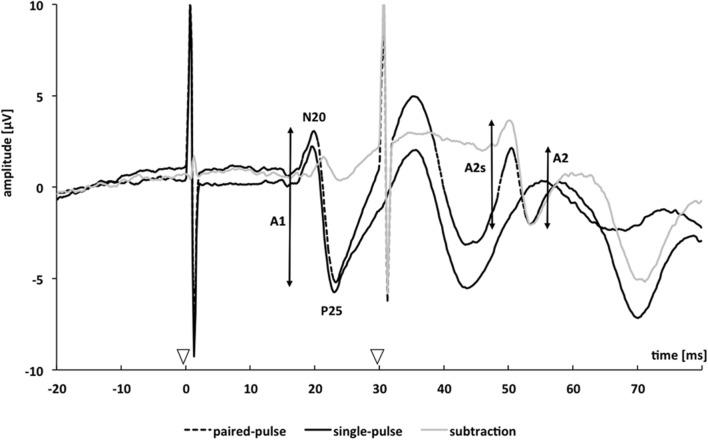
**Schematic representation of paired-pulse somatosensory evoked potentials from one single subject.** Somatosensory evoked potentials are measured over CP3 or CP4 after single (continuous black trace) and paired-pulse stimulation with a stimulus-onset asynchrony of 30 ms (dotted black trace). The continuous grey trace resulted by subtracting the single-pulse trace from the paired-pulse trace. The analyzed amplitudes of the first response (A1) and second response (A2) after paired-pulse stimulation are marked by vertical bars; amplitudes of the second response after subtracting the response to a single pulse are denoted as A2s. Arrowheads mark onsets of the applied electrical stimuli.

We applied a paired-pulse protocol consisting of paired electrical stimulation of the median nerve with a SOA of 30 ms in combination with recordings of SEPs. Details of the described methods have also been published elsewhere (Höffken et al., 2007, 2010, 2013; Lenz et al., 2011, 2012): “Nerve stimulation (pulse duration 0.2 ms, repetition rate of the paired stimuli 2 Hz) was performed using a block electrode placed on the wrist of the left and right hand. Single-pulse SEP (spSEP) of both hands were recorded additionally using the same setup, alternating with the paired-pulse SEP (ppSEP; left spSEP, left ppSEP, right spSEP, and right ppSEP). Subjects had to report a prickling sensation in the thumb, index, and middle finger of the stimulated hand to verify correct positioning of the stimulating block electrode. Stimulation intensity was individually adjusted to the 2.5-fold of individual sensory thresholds. Median nerve stimulation at individually adjusted intensity induced a small muscular twitch in the thenar muscles. During median nerve stimulation and SEP recordings, subjects were seated in a comfortable chair and were instructed to relax but stay awake with closed eyes. SEP signals were amplified and filtered using a BrainAmp magnetic resonance amplifier (Brain Products GmbH, Gilching, Germany) and digitized in a PC running the BrainVision Recorder software package (Brain Products GmbH). Paired-pulse SEP recordings were done using a 3-electrode array. Two electrodes (CP3 and CP4) were located over the left and right primary somatosensory cortex (S1), 2 cm posterior to C3 and C4 according to the 10–20 EEG international system (Jasper, 1958). A reference electrode was placed over the midfront (FZ) position. The electrical potentials were recorded in epochs from 0 to 200 ms after stimulus onset. A total of 200 stimulus-related epochs were recorded at a time for single and paired stimuli on each side. Offline, SEP raw data were segmented and baseline corrected, movement and muscle artifacts (amplitudes ≥ 100 μV) were rejected, and averaging was performed (see also Lenz et al., 2012). Peak-to-peak amplitudes of the cortical N20/P25 response component for the first and second paired-pulse stimulus were analyzed (Höffken et al., 2007, 2010, 2013; Lenz et al., 2011, 2012).” As exemplarily shown in **Figure 1**, after paired-pulse stimulation the response to the second pulse rides on the response to the first pulse, leading to a superimposition of both evoked potentials. Therefore, the amplitude of the response to the second pulse may misleadingly appear to be higher or lower. To assess “true” paired-pulse interaction, linear superposition effects had to be factored out by subtracting the response to the single-pulse stimulation from the paired-pulse stimulation trace. We analyzed the second ppSEP amplitude after linear subtraction of the spSEP (A2s) and referred it to the first ppSEP amplitude before linear subtraction (A1). PPS was expressed as a ratio (A2s/A1) of the amplitudes of the second (A2s) and the first (A1) peak (Höffken et al., 2010; Lenz et al., 2011; see **Figure 1**).

### Transcranial Direct Current Stimulation

**FIGURE 2:**
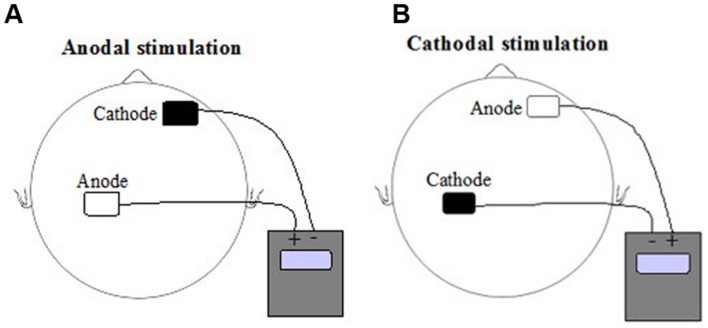
**Schematic representation of current direction and electrode positions for **(A)** anodal transcranial direct current stimulation (tDCS) and **(B)** cathodal tDCS of left hemisphere somatosensory cortex**.

Transcranial direct current stimulation was delivered by a battery-driven constant DC current stimulator (NeuroConn, Ilmenau, Germany) using a pair of rubber electrodes in a 5 cm × 7 cm (surface 35 cm^2^) 0.9% saline – soaked synthetic sponge. The electrodes were placed according to the 10–20 EEG international system (Jasper, 1958). The stimulation electrode (to which the terms anodal and cathodal are applied) was placed over CP4, whereas the reference electrode was positioned over the contralateral orbita (see **Figure 2**). The subjects were blind with regard to the type of stimulation (anodal, cathodal, or sham). The experimenter received a 5-digit number from the main investigator that encoded the type of stimulation (so called “study mode” of the NeuroConn tDCS device). So, neither the experimenter nor the subject could know the type of DC stimulation (anodal, cathodal, or sham). The current was applied for approximately 20 min (1170 s, plus 15 s at the beginning and at the end of stimulation, when the current was ramped up and down) with an intensity of 1.0 mA (current density 0.029 mA/cm^2^, total charge 0.33 C/cm^2^). A voltmeter integrated in the DC stimulator controlled constant current flow. Actual voltage, current and impedance were shown on the display and could be controlled by the experimenter. Using a ramp-like switch, current strength of tDCS gradually increased for the first and decreased for the last 15 s. During the sham condition current flowed for a period of 30 s at the beginning of stimulation and then turned off. This procedure induced a weak-prickling sensation, so stimulation condition was indistinguishable.

### Experimental Setting

The study was performed as a randomized, double-blinded and controlled trial. Each group consisted of 12 subjects, balanced to age and gender (**Table 1**). Electrophysiological measurements were assessed immediately prior and after transcranial stimulation.

**Table 1:** Distribution of subjects in each group.

	Anodal	Cathodal	Sham
*n* (total)	12	12	12
Mean age	26.6 years	27.0 years	28 years
SD age	7.4 years	8.4 years	7.7 years
*n* (female)	5	7	6
*n* (male)	7	5	6


### Statistics

Paired-pulse SEP ratios of the three groups (anodal, cathodal, or sham tDCS) were compared using analysis of variance (ANOVA) for repeated measurements with “side” and “time” as within-subject factors and “stimulation group” as between-subject factor. Factor “side” means “stimulated hemisphere” vs. “non-stimulated hemisphere,” and factor “time” is “pre measurement” vs. “post measurement.” *Post hoc t*-tests were conducted to confirm ANOVA results. Statistical analyses were performed using the IBM SPSS Statistics 23 software package.

## Results

**FIGURE 3:**
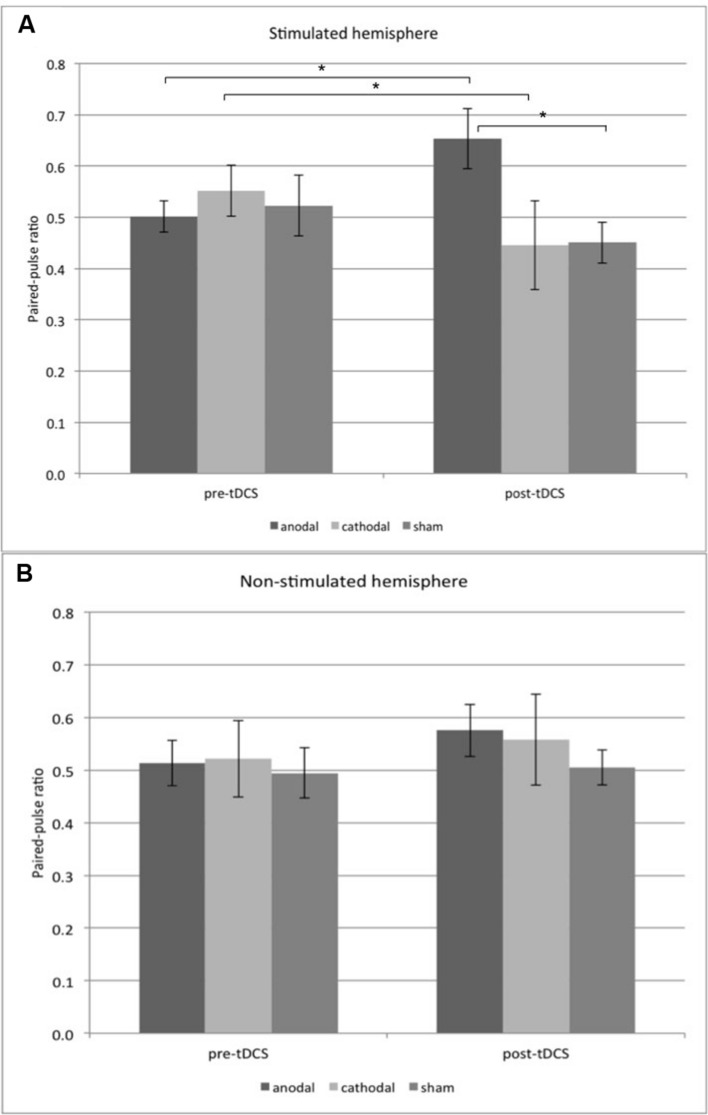
**Mean paired-pulse ratios ± SD are plotted for the stimulated **(A)** and non-stimulated **(B)** hemisphere.** Pre-tDCS and post tDCS ratios are shown for the anodal, cathodal, and sham group. In the stimulated hemisphere, paired-pulse ratios after anodal tDCS are increased compared to pre-tDCS. After cathodal tDCS, paired-pulse ratios are decreased compared to pre-tDCS (student paired *t*-test, *p* < 0.05). Post-tDCS ratios are increased in the anodal group compared to the sham group (student unpaired *t*-test, ^∗^*p* < 0.05). tDCS, transcranial direct current stimulation.

**Table 2** shows all mean amplitude ratios and standard deviations. ANOVA revealed no significant effects of the within-subject factors “side” [*F*(1,33) = 0.114; *p* = 0.737] and “time” [*F*(1,33) = 0.276; *p* = 0.603] and the between-subject factor “stimulation group” [*F*(2,33) = 0.507; *p* = 0.607]. Also, we found no significant interactions of the factors “side ^∗^ group” [*F*(2,33) = 1.062; *p* = 0.357], “side ^∗^ time” [*F*(1,33) = 1.600; *p* = 0.215] and “time ^∗^ group” [*F*(2,33) = 3.119; *p* = 0.057]. In contrast, ANOVA revealed an interaction of the three factors “side ^∗^ time ^∗^ group” [*F*(2,33) = 3.695; *p* = 0.036]. *Post hoc t*-tests showed increased amplitude ratios in the anodal group compared to the sham group (Student unpaired *t*-test, *p* = 0.011, d_Cohen_ = 1.346) in the stimulated left hemisphere after tDCS stimulation (see **Figure 3** and **Table 2**). There was no significant difference between anodal and cathodal post tDCS values (*p* = 0.062). For the stimulated left hemisphere, we found increased amplitude ratios post tDCS compared to pre tDCS in the anodal group (Student paired *t*-test, *p* = 0.014, d_Cohen_ = 0.895) and decreased amplitude ratios post tDCS compared to pre tDCS in the cathodal group (*p* = 0.048, d_Cohen_ = -0.41; see **Figure 3** and **Table 2**). We found no differences in the sham group.

**Table 2:** Mean amplitude ratios and statistics.

	Anodal tDCS		Cathodal tDCS		Sham tDCS	
			
	Pre	Post	Pre	Post	Pre	Post
Ratio A2s/A1 [mean ± SD]	0.5 ± 0.11	0.65 ± 0.21	0.55 ± 0.17	0.45 ± 0.3	0.52 ± 0.2	0.45 ± 0.14
Paired *t*-test, pre vs. post [*p*]	0.014		0.048		0.173	
Unpaired *t*-test, post tDCS anodal vs. sham [*p*]					0.011	


*Ratio A2s/A1 = ratio of the second divided by the first N20-P25 amplitude after paired-pulse stimulation, after linear subtraction of pp A1; tDCS, transcranial direct current stimulation.*

## Discussion

The present study investigated whether anodal and cathodal tDCS modulate the excitability of the human primary somatosensory cortex in the same polarity dependent manner as it was previously described for the motor and visual cortices. Paired-pulse stimulation in combination with SEP recordings as a well-established marker of somatosensory cortical excitability were used (Höffken et al., 2007, 2010, 2013; Lenz et al., 2011, 2012). After anodal tDCS, PPS was reduced in the ipsilateral S1 compared to sham stimulation, indicating an excitatory effect of anodal tDCS. In contrast, PPS in the stimulated left hemisphere was increased after cathodal tDCS, indicating an inhibitory effect of cathodal tDCS. Sham stimulation induced no pre–post differences. The results of our study are in line with those of previous studies from motor and visual cortices, in which anodal stimulation has been shown to enhance cortical excitability while conversely, cathodal stimulation decreases it (Nitsche and Paulus, 2000, 2001; Antal et al., 2003; Nitsche et al., 2003a). Thus, the previously reported sensory changes after tDCS, like changes in tactile discrimination (Rogalewski et al., 2004; Ragert et al., 2008), thermal perception (Grundmann et al., 2011) and pain perception (Antal et al., 2008) might in fact be explained by excitability changes in S1. Based on this knowledge, experimental designs in order to modulate somatosensation using tDCS, for example in elderly people and pain patients, can be selected accordingly. For example, in one recent study anodal tDCS was used to enhance motor performance in elderly people (Zimerman et al., 2013). In a previous study, we found reduced PPS in elderly people, which correlated with impaired tactile discrimination skills (Lenz et al., 2012). Presuming that disinhibition in elderly is not due to compensatory effects, cathodal tDCS in order to enhance PPS might be helpful to improve tactile discrimination in those people. Recently, a systematic review was published which does not support the idea that tDCS has a reliable neurophysiological effect beyond MEP amplitude modulation (Horvath et al., 2014). The results of our study contradict the results of this report, as we found tDCS induced cortical excitability changes in S1. As M1 and S1 are localized next to one another on the cortical surface and the size of our tDCS electrodes was 5 cm × 7 cm, it cannot be ruled out that the motor cortex was also stimulated. Kaneko et al. (1994a,b) described corticocortical projections between S1 and M1. Furthermore, Schabrun et al. (2012) demonstrated a positive correlation between S1 and M1 excitability after sensory peripheral electrical stimulation, suggesting that our tDCS induced effect were not only restricted to S1, but also to M1. Thus, we cannot completely rule out the possibility that the measured PPS changes in S1 are at least partly induced by M1 stimulation. But, in our study, we analyzed peak-to-peak amplitudes of the cortical N20/P25 component, which is clearly a SEP component (Lueders et al., 1983; Allison et al., 1989; Namiki et al., 1996). Previous studies showed differences in cortical excitability during the menstrual cycle (Inghilleri et al., 2004; Hausmann et al., 2006). Unpublished data of our group could not show any effect of different stages of the menstrual cycle and serum concentration of gonadotropic hormones on PPS behavior of median nerve SEPs. If menstrual cycle influences the effect size of tDCS itself has not been assessed yet, and cannot be ruled out as a possible interfering factor. In our recent study, changes of excitability were assessed in both hemispheres (stimulated vs. non-stimulated hemisphere). We observed significant tDCS-induced effects only in the stimulated S1 but not contralateral. This finding is in line with a tDCS-study by Lang et al. (2004). They showed that unilateral tDCS over M1 did not induce effects in contralateral M1 by affecting transcallosal inhibition (Lang et al., 2004). Similar effects can be postulated for S1, but were not investigated explicitly in our study. It has to be noted that bilateral effects of unilateral tDCS might depend on investigated and stimulated regions. Whereas, Lang et al. (2004) stimulated over the cortical representation area of the hand, Zhao et al. (2015) stimulated over the suprahyoid/submental representation in M1 and observed bilateral effects in a subgroup (Zhao et al., 2015).

Despite substantial experimental and theoretical work, the mechanisms mediating paired-pulse behavior in the human S1 are not fully understood. There is agreement that presynaptic mechanisms play a crucial role (Hashimoto and Kano, 1998; David-Jürgens and Dinse, 2010). In the cat visual cortex, suppression is more consistent with thalamocortical synaptic depression than with inhibition (Carandini et al., 2002; Freeman et al., 2002). In addition, there is evidence that GABA_B_ receptors seem to be involved in regulation of PPS (Porter and Nieves, 2004). In addition to the contribution of GABAergic mechanisms, there is evidence for the involvement of glutamatergic transmission in the paired-pulse phenomenon (Takahashi et al., 1996; von Gersdorff et al., 1997). Because of differences in the PPS between cortical and thalamic cells, it has been argued that inheritance of thalamic response properties is unlikely to account for long-lasting forward suppression (Wehr and Zador, 2005). For human subjects, based on multichannel SEP recordings after paired median nerve stimulation, it has been shown that PPS is generated at least rostral to the brainstem nuclei (Höffken et al., 2010). Matsunaga et al. (2004) investigated the effect of sensorimotor tDCS on different SEP components. They found significantly increased amplitudes of the P25/N33, N33/P40 and P22/N30 component, but no effect on N20/P25 amplitudes. In our study, we did not analyze raw N20/P25 amplitudes, but the ratios of the amplitudes of the second and the first N20/P25 peak after paired-pulse stimulation. Our raw amplitudes also showed no significant differences before and after tDCS (data not shown). The aim of our study was to investigate excitability changes in S1. Thus, we compared paired-pulse ratios instead of raw amplitudes, which are a well-established marker of somatosensory cortical excitability (Höffken et al., 2007, 2010, 2013; Lenz et al., 2011, 2012). In a previous study, our working group successfully used PPS to show that high-frequency (5 Hz) repetitive transcranial magnetic stimulation applied over the left S1 evokes sustained excitability enhancement in the ipsilateral cortex (Ragert et al., 2004), just as it had been described before for the motor cortex (Pascual-Leone et al., 1994).

Compared with other non-invasive brain stimulation techniques, tDCS is tolerated well and has only few contraindications. However, there are some concerns about study conceptions and mechanistic models. For example, several parameters and variables have influence on tDCS efficacy and outcome. There are physical markers like current density, total charge and impedance that directly have effects on stimulation. Electrode position, stimulation intensity, hair thickness, and skin conduction are other parameters (see Nitsche et al., 2008; Horvath et al., 2014 for review). Moreover, the inter- and intraindividual variability of tDCS effects, electrophysiological and psychophysiological parameters have a high impact on study results and their interpretation. In the recent literature, physical parameters like electrode position, current density and stimulation duration vary from study to study (Nitsche et al., 2008; Horvath et al., 2014). In the present study, we applied a commonly used current density of 0.029 mA/cm^2^ for about 20 min. This combination has been used in several studies (see Nitsche et al., 2008 for review). Electrode positions over CP3 and CP4 (10–20 EEG international system; Jasper, 1958) have been chosen according to electrode positions for SEP measurements over S1.

The results of our study show that tDCS can be used to modulate the excitability of S1 in polarity-dependent manner, which can be assessed by PPS. An interesting topic for further studies could be the investigation of direct correlations between sensory changes and excitability changes induced by tDCS. Furthermore, in this study, lasting-after effects have not been investigated. So, studies on the duration of induced cortical effects on paired-pulse behavior could give more insight into the neuroplastic processes and consolidation.

## Author Contributions

RR: study conception and design, acquisition and analysis of the data, drafted the manuscript; MS-K: study conception and design, acquisition and analysis of the data, drafted the manuscript; ML: study conception and design, acquisition and analysis of the data, drafted the manuscript; TG: study conception and design, acquisition of the data, analysis of the data, critical revision of the manuscript; AS: study conception and design, acquisition of the data, analysis of the data, critical revision of the manuscript; PS: study conception, critical revision of the manuscript; MT: study conception, critical revision of the manuscript; OH: study conception, analysis of the data, critical revision of the manuscript. All authors read and approved the final manuscript.

## Conflict of Interest Statement

The authors declare that the research was conducted in the absence of any commercial or financial relationships that could be construed as a potential conflict of interest.
